# Distinct Effects of Milks From Various Animal Types on Infant Fecal Microbiota Through *in vitro* Fermentations

**DOI:** 10.3389/fmicb.2020.580931

**Published:** 2020-09-10

**Authors:** Na Li, Bailiang Li, Jiaqi Guan, Jialu Shi, Smith Etareri Evivie, Li Zhao, Guicheng Huo, Song Wang

**Affiliations:** ^1^Key Laboratory of Dairy Science, Ministry of Education, Northeast Agricultural University, Harbin, China; ^2^College of Food Sciences, Northeast Agricultural University, Harbin, China; ^3^Food Science and Human Nutrition Unit, Department of Animal Science, Faculty of Agriculture, University of Benin, Benin City, Nigeria

**Keywords:** breastfeeding, mare milk, fermentation, fecal microbiota, gas pressure, pH

## Abstract

Human milk is compatible with infant intestinal microbiota and is vital for infant health. However, most infants do not receive sufficient exclusive breastfeeding, and the effects of including other types of animal milk on the gut microbiota of infants are unclear. Therefore, the objective of this study was to elucidate the impact of milk from various animal sources on infant fecal microbiota through *in vitro* fermentation. The types of milk assessed include cow milk, goat milk, camel milk, mare milk, human milk, and infant formula milk. Here we determined the gas pressure, pH, and microbiota after 24 h fermentation. Results showed that mare milk had the lowest gas pressure rating, with levels similar to human milk. More so, pH analysis demonstrated that other milk types were identical to human milk. Bacterial 16S rRNA gene sequence analysis revealed that all milk types increased the abundance of *Bifidobacterium* and *Lactobacillus*, which was proportional to the lactose content of milk. Moreover, mare milk also significantly increased the relative abundance of *Akkermansia*. Collectively, results from mare milk (gas pressure, pH, and microbiota) were comparable to that of human milk, and thus support the theoretical basis for exploring the development of a mare milk-based infant formula.

## Graphical Abstract

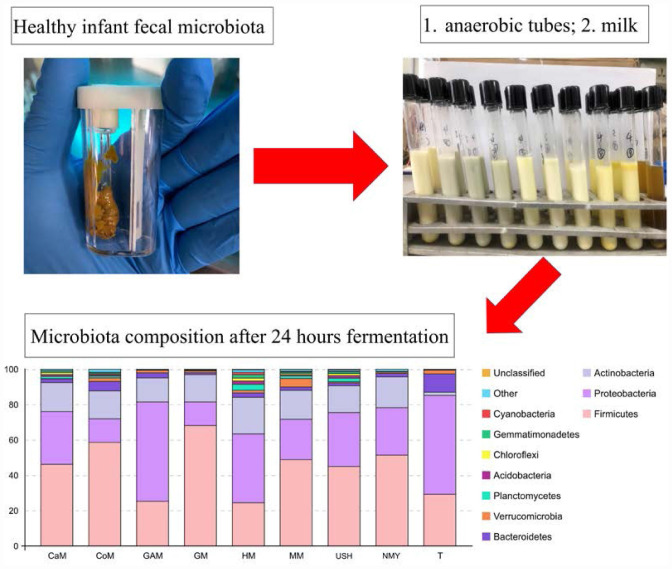

## Introduction

Milk is an essential food for infants, especially human milk, which is rich in protein, fat, lactose, and a variety of vitamins. Based on its immense nutritional value, it has arguably been called “nature’s nearly most perfect food.” As compared to non-breast milk-fed or formula-fed infants, human milk is highly effective against diarrhea morbidity and mortality (Lamberti et al., 2011) and decreases symptoms of asthma (Klopp et al., 2017), inflammatory bowel disease (Xu et al., 2017), and obesity (Forbes et al., 2018). Some posit that human milk exerts these desirable properties in part because of its effects on infant intestinal microbiota. Globally, the breastfeeding rate is around 37%, but in China, it is barely 20.8%, and this is currently on the decline. When breastfeeding is inadequate or impossible, safe and nutritious substitutes must serve as replacements. While infant formulas compositions are closely similar to that of human milk, formula-fed infant gut microbiota composition is still markedly different from those of breastfed infants (Backhed et al., 2015). It thus follows that studies into developing an infant milk formula similar to human milk in terms of specific parameters such as microbiota structure, gas pressure and pH value, become imperative.

Cow milk is a rich and inexpensive source of protein and calcium and is known for the prominent role it plays in preventing and treating moderate and severe malnutrition in children (Agostoni and Turck, 2011). Interestingly, mare milk has attracted people’s research attention as a substitute to cow milk because of its nutritional values as well as its unique properties as a formula for children allergic to cow milk (Businco et al., 2000). Research efforts into the possible use of goat milk have increased around the world too because it is highly nutritious, healthy for use in treating many gastrointestinal diseases, more comfortable to digest, less allergenic than cow milk and similar to human milk, it has been the first choice for infants (Coveney and Darntonhill, 1985; Laravilloslada et al., 2004). Khalesi reported that camel milk was used as biomedicine to treat several health conditions in some arid rural communities of Asia and Africa, such as asthma, edema, and diabetes (Khalesi et al., 2017). The nutritional values of camel milk are well known despite limitations reported by Mihic (Mihic et al., 2016). Thus, cumulative research findings suggest that cow milk, goat milk, camel milk, and mare milk all have the potential to replace human milk at least partially.

The present study compared the contents of main components of cow milk, goat milk, camel milk, mare milk, human milk, and infant formula milk, and compared the fermentation effect of these kinds of milk using an *in vitro* model. To our knowledge, this is the first *in vitro* fermented research examining the impact of milk from various animal types. This study aimed to compare these kinds of milk through gas pressure, pH, and microbiota compounds detected in anaerobic tubes after 24 h. Also, the relationship between milk types, simulated infant microbiota, gas pressure, and pH values was illustrated by CCA analysis, which was used to explore the association between multiple variables. It is anticipated that, based on these analyses, potential milk replacements for human milk would be achieved, which could then be adopted for use in countries where it may not be entirely convenient to give exclusive breastfeeding.

## Materials and Methods

### Milk Collected From Various Types of Animals

Seven milk types were selected for this research, and the control was the Gifu anaerobic medium (GAM broth; Qingdao Hope Bio-Technology Co., Ltd., China) (Gotoh et al., 2017). Breast milk samples were collected from ten 25-year-old healthy mothers 3 months after vaginal delivery at term (Gestational age 38.0–41.0 weeks). Human milk samples were collected from mothers who received no antibiotics and probiotics supplements 3 months before sampling. Also, written informed consent was obtained from the guardians of all infants involved in this study. To collect milk samples, we washed the breast with the sterile water, swabbed the nipple and areola with An’erdian R type III skin antiseptic solution (0.5% (w/v) available iodine and 0.1% (w/v) chlorhexidine gluconate) (LiKang, Shanghai, China), and last swabbed with sterile water. The infant formulas NMY and USH were purchased from the market [Walmart (China) Investment Co., Ltd., China], and stored at room temperature. The four milk types – cow milk (CoM), goat milk (GM), camel milk (CaM) and mare milk (MM) – were collected from 15 Holstein cows (Heilongjiang Province, China), 15 Chinese milk goats (Heilongjiang Province, China), 20 Bactrian camels (Hebei Province, China) and 20 mares (Inner Mongolia, China), respectively. Milk from various types of animals was collected into sterile tubes after discarding the first drops and was frozen at −20°C immediately upon collection. Before transportation for analyses, sterile tubes were stored in dry ice at −80°C at the Northeast Agricultural University storage facility for 3 days at the most. Aliquots were kept at −80°C for further study.

### Main Components of Milk Detection

The total solid content of each milk type was estimated by the drying method (ISO 6731: 2010); the fat content by the Gerber method (ISO 488: 2008); the content of lactose by high-performance liquid chromatography (HPLC) (ISO 22662: 2007); the protein content by the Kjeldahl method (ISO 8961-1: 2014).

### Donor Information and Fecal Collection

Fecal samples from ten healthy full-term exclusively breastfed infants were collected under anaerobic conditions. Infants included five individuals from natural birth and five individuals from cesarean section (Gestational age 39.0–40.2). Infants did not receive any antibiotic treatment and did not have any known gastrointestinal illnesses (Supplementary Table S1).

Fecal samples were collected within 1 h before the commencement of fermentation anaerobically and homogenized immediately afterward. This study was ratified by the Ethical Committee of Northeast Agricultural University, which is following the Declaration of Helsinki.

### Fermentation and Determination of Gas Pressure and pH

The different kinds of milk samples were fermented with infant gut microbiota using an *in vitro* model to simulate the composition of the microbiota of the infant distal colon as described previously by Koecher et al. (2014). Milk samples (10 mL) were prepared, adjusted to, and maintained at pH 2.0. After 15 min of incubation in a water bath (model, country) at 37°C, 2% pepsin (w/w, based on the volume of milk) was added and then was incubated in the water bath and hydrolyzed for 2 h. Then we adjusted pH to 7.5 with 2 M NaOH and 2% (w/w) trypsin was added to the solution for the second step digestion. Similarly, hydrolysis was carried out in the water bath (model country) at 37°C for 2 h, and the digestion process ended by enzyme inactivation in boiling water for 10 min, and transferred to the 20 mL anaerobic tubes, capped, and incubated.

After the collection, samples were mixed with PBS solution (6:1). The obtained mixture was prepared using reducing solution (380 mL DD H_2_O, 2.52 g cysteine hydrochloride, 16 mL 1 N NaOH, 2.56 g sodium sulfide non-anhydride) at a 2:15 ratio (Carlson et al., 2017). 2 mL of the prepared fecal inoculum was added to each of the anaerobic tubes, flushed with N_2_, sealed, and then placed in a 37°C constant temperature incubator immediately for 24 h. Gifu anaerobic medium was in the same conditions to compare with milk. Samples were analyzed after 24 h. Gas pressure was measured in each anaerobic bottle using a manometer (Hangzhou Halo Medical Technology Co., Ltd., China), and pH was detected at the same time using a pH meter (Shanghai Precision Scientific Instrument Co., Ltd., China). Then 1 mL of copper sulfate (200 g/L) was added into the tubes to stop the fermentation process. Immediately, samples were stored at −80°C for DNA extraction.

### Analysis of Microbiota

Genomic DNA was extracted from samples using the E.Z.N.A. stool DNA Kit (Omega Biotek, Norcross, GA, United States) following manufacturer’s protocols. The first PCR amplified the V3–V4 region of the 16S rRNA gene using the 341F and 806R primers: 5′-CCTACGGGNGGCWGCAG-3′ (forward primer) and 5′- GGACTACHVGGGTATCTAAT -3′ (reverse primer). Purified amplicons were pooled in equimolar and paired-end sequenced (2 × 250) on an Illumina platform. The details of DNA extraction, PCR amplification, quantitative PCR, amplicons purification, sequencing, and high-quality sequences criteria are those described by Li (Li et al., 2020).

Raw reads were further filtered to get high-quality clean reads according to the following rules: removing reads containing more than 10% of unknown nucleotides, and removing reads containing less than 80% of bases with quality (*Q*-value) >20. Paired-end clean reads were merged as raw tags using FLSAH (Magoč and Salzberg, 2011) (v 1.2.11) with a minimum overlap of 10 bp and mismatch error rates of 2%. Noisy sequences of raw tags were filtered with QIIME (V1.9.1) (Caporaso et al., 2010) pipeline under specific filtering conditions (Bokulich et al., 2013) to obtain the high-quality clean tags. Valid tags were clustered into operational taxonomic units (OTUs) of ≥ 97% similarity by UPARSE (Edgar, 2013) pipeline. The tag sequence with the highest abundance was selected as a reprehensive sequence within each cluster. Abundance statistics of each taxonomy were constructed in a Perl script and visualized by SVG software.

To validate the complexity of species diversity of samples, the alpha diversity analysis Chao1, Simpson, ACE, and Shannon index were calculated in QIIME (V1.9.1). OTU rarefaction curve and rank abundance curves were also plotted in QIIME. The Tukey HSD test was used to calculate the statistical significance of alpha index comparison among groups. The species abundance of microorganisms at the phylum, family, and genus levels were compared. Furthermore, we used linear Discriminant Analysis Effect Size (LEfSe) to identify the potential microbial biomarkers related to milk from various animal types with the threshold value of 2 (Segata et al., 2011).

### Canonical Correspondence Analysis

Canonical correspondence analysis (CCA) model was established to analyze the correlation between gas pressure and pH with microbiota at the genus level by the envfit test conducted within the R vegan package using the R vegan package.

### Statistics Analysis

A minimum of three independent experiments was performed for each assay. All values were expressed as the mean ± standard deviation (SD). Analysis of the data was carried out using SPSS 20.0 software (SPSS Inc., Chicago, IL, United States) and GraphPad Prism 7.00 for Windows (GraphPad Software, La Jolla, CA, United States). The statistical significance of data comparisons was determined using a one-way analysis of variance (ANOVA), followed by Tukey HSD test and Tukey B. Values of *p* < 0.05 were judged to be statistically significant.

## Results

### The Contents of the Main Components of Different Milk Types

The main components of milk from various types of animals were determined (Table 1). The result showed that camel milk and cow milk were higher in protein compared to other kinds of milk. Camel milk, infant formula powder USH, and goat milk were higher in fat than different milk types. The content of lactose was higher in mare milk, human milk, and infant formula NMY and USH. Compared to other milk types, the goat milk and camel milk were significantly higher in total solid contents.

**TABLE 1:** The content of the main component of different kinds of milk (%).

	CoM	GM	CaM	MM	HM	NMY	USH
Protein	3.260.06^f^	2.980.04^e^	3.500.03^g^	2.630.04^d^	2.470.04^c^	1.470.01^a^	1.670.01^b^
Fat	3.520.03^c^	4.170.14^e^	5.610.02^g^	1.770.02^a^	3.420.03^b^	3.680.01^d^	4.520.01^f^
Lactose	4.510.02^b^	3.950.03^a^	5.090.02^c^	6.240.06^f^	6.820.02^e^	6.140.01^de^	6.040.01^d^
Total solid	11.570.04^a^	15.880.04^e^	16.480.03^f^	12.430.05^c^	12.910.02^d^	11.790.01^b^	12.370.01^c^

*All data are presented as mean ± standard deviation (SD). Tukey indicates significant differences (*p* < 0.05) among different treatments with different letters (a,b,c,d,e,f,g). CoM, cow milk; GM, goat milk; CaM, camel milk; MM, mare milk; HM, human milk; NMY, infant formula powder NMY; USH, infant formula powder USH.*

### Gas Pressure of All Fermented Groups

**FIGURE 1:**
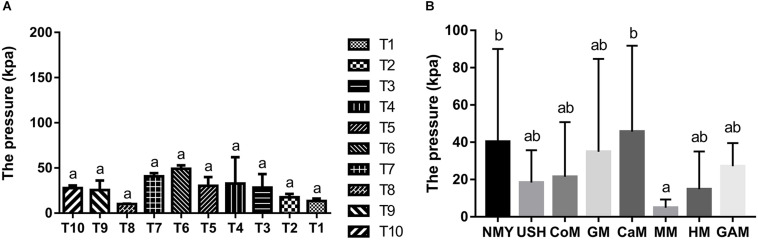
Gas pressure of the ten infant fecal slurry samples fermented with the Gifu anaerobic medium **(A)**, and Gas pressure of fermented groups **(B)**. Data are represented as mean ± SD. ^a,b^Means with different letters differ significantly among the groups (*p* < 0.05). NMY and USH, infant formula powder; CoM, cow milk; GM, goat milk; CaM, camel milk; MM, mare milk; HM, human milk; GAM, Gifu anaerobic medium.

The gas pressure of the ten infant fecal slurry samples fermented with the Gifu anaerobic medium and gas pressure of the eight groups are presented in Figure 1. There was no significant difference in the fermentation of the Gifu anaerobic medium in the fecal slurry of ten infants (*p* > 0.05). It was demonstrated that the 10 fecal slurries had similar gut microbiota composition (Figure 1A). After 24 h of fermentation, the group which fermented with camel milk had the highest gas pressure (45.68 k Pa) and was similar to the group which fermented with infant formula NMY (40.32 k Pa). The gas pressure of the group which fermented with infant formula USH (18.34 k Pa) and the group which fermented with cow milk (21.48 k Pa) was much more similar to the group which fermented with human milk (14.76 k Pa). And there was no significant difference between the effect of mare milk on gas pressure and human milk through the fermentation (Figure 1B).

### pH Measurement of Fermented Groups

**FIGURE 2:**
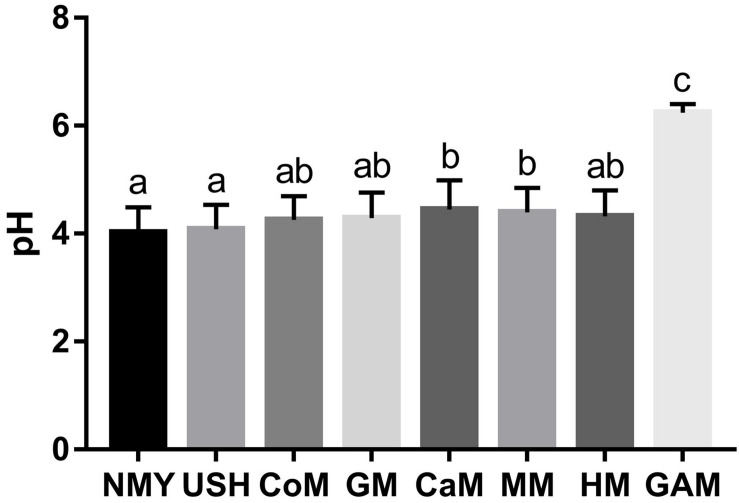
pH of fermented groups. Data are represented as mean ± SD. ^a– c^Means with different letters differ significantly among the groups (*p* < 0.05). NMY and USH, infant formula powder; CoM, cow milk; GM, goat milk; CaM, camel milk; MM, mare milk; HM, human milk; GAM, Gifu anaerobic medium.

The pH of the eight groups is presented in Figure 2. After 24 h of fermentation, the group which fermented with Gifu anaerobic medium had the highest pH value and was significantly different from other groups (*p* < 0.05). The group which fermented with infant formulas NMY and USH had a lower pH value and were substantially different from the group, which fermented with camel milk and mare milk (*p* < 0.05). These showed clearly that infant formulas were better at producing acid than the other milk types. Human milk was not significantly different from other milk types (*p* > 0.05). Interestingly, results also indicated that the effect of mare milk on pH was similar to human milk.

### Gut Microbiota Compositions

**FIGURE 3:**
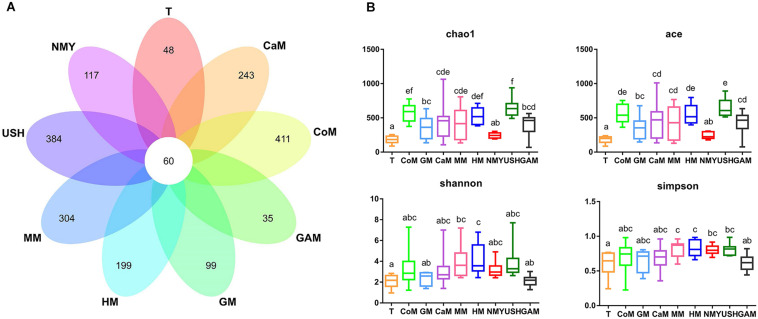
**(A)** Venn diagram of OTUs; **(B)** Four metrics of analysis for alpha-diversity among all groups. T, initial infant fecal slurry of ten infants; NMY and USH, infant formula powder; CoM, cow milk; GM, goat milk; CaM, camel milk; MM, mare milk; HM, human milk; GAM, Gifu anaerobic medium.

We analyzed the V3-V4 regions of the bacterial 16S rRNA gene sequence to characterize the microbial communities present in samples. A total of 12,965,663 (mean, 145,681; range, 70,095–225,555) bacterial tags, of which 12,747,505 (mean, 143,230; range, 68,951–224,537) passed quality filters. Species accumulation and rarefaction curves (Supplementary Figure S1) of all samples also validated the accuracy of the sampling process. Following the taxonomic assignment, 60 of the 3219 OTUs obtained were shared among all groups. Additionally, we found that most OTUs were uniquely present in different groups (Figure 3A). In particular, the groups which fermented with cow milk, mare milk, camel milk, human milk, and infant formula USH all had unique OTUs.

As expected, different kinds of milk and Gifu anaerobic medium were strongly associated with the richness index (Chao1 and ACE) and diversity index (Shannon and Simpson) of intestinal microbiota (Figure 3B). Compared to the initial infant fecal slurry (T group), the richness of microbiota of all kinds of milk and Gifu medium increased significantly except the infant formula NMY, with the groups which fermented with cow milk, camel milk, mare milk, and infant formula USH appearing more similar to the group which fermented with human milk. The diversity of microbiota in all groups showed no significant difference among the groups which fermented with human milk, cow milk, camel milk, mare milk, and infant formula USH. These findings suggest that cow milk, camel milk, mare milk, and infant formula USH were potential replacements of human milk.

### Specific Microbial Phyla and Families Abundant Among Milk From Various Types of Animals

**FIGURE 4:**
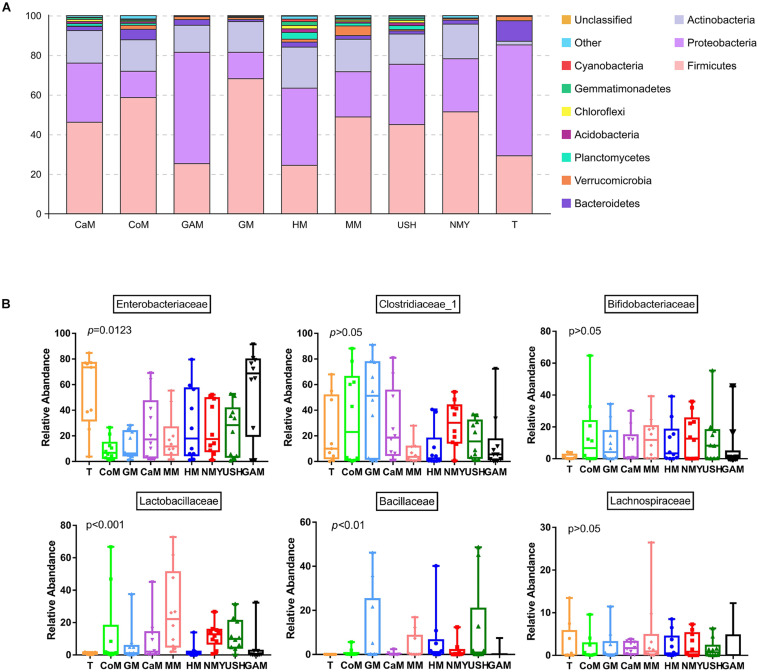
Microbial relative abundance in different kinds of milk fermented with infant fecal slurry. The capital letters on the various graphs signify: **(A)** phyla level; **(B)** dominant bacteria of a family level. T, initial infant fecal slurry of ten infants; NMY and USH, infant formula powder; CoM, cow milk; GM, goat milk; CaM, camel milk; MM, mare milk; HM, human milk; GAM, Gifu anaerobic medium.

Almost all phyla and families showed disproportional abundance across different groups, and milk from various animal types was related to specific taxa. As shown in Figure 4, 32 phyla were identified in all groups. Most of the sequences are mainly composed of five phyla, namely, Firmicutes, Proteobacteria, Actinobacteria, Bacteroidetes, and Verrucomicrobia. Compared with the T group, the relative abundance of Firmicutes in most groups was significantly increased; however, the groups which fermented with Gifu anaerobic medium and human milk were lower than the T group. It was also observed that the relative abundance of Proteobacteria in most groups significantly decreased, except for the group which fermented with Gifu anaerobic medium. The highest relative abundance of Actinobacteria was noticed in the group which fermented with human milk. Besides, the relative abundance of Bacteroidetes in all fermented groups was significantly decreased compared to the T group. More so, the relative abundance of Verrucomicrobia (mainly *Akkermansia*) in most groups (including the T group) was significantly decreased, except the group fermented with mare milk. Compared with the group which fermented with human milk, the relative abundance of Firmicutes in other milk fermented groups were significantly increased, while the relative abundance of Proteobacteria and Actinobacteria in other fermented groups were significantly decreased. After 24 h fermentation, the effects of mare milk and infant formula USH were more similar to human milk through the comparative sum of the dominant phyla (Figure 4A).

Taxa analyses showed that 199 bacterial families were detected in the milk samples. Samples were dominated by Enterobacteriaceae, Clostridiaceae_1, Bifidobacteriaceae, Lactobacillaceae, Bacillaceae, and Lachnospiraceae groups (Figure 4B). Most taxa were similarly abundant in groups, while the relative abundances of families were different. Compared to the T group, the abundance of Bifidobacteriaceae, Lactobacillaceae, and Bacillaceae in all groups increased. Furthermore, more abundant in Bifidobacteriaceae was detected in the groups which fermented with mare milk and infant formula NMY, while highest Lactobacillaceae abundance was found in the group which fermented with mare milk. The Bacillaceae family was predominant in the group, which fermented with infant formula USH while compared to the other groups. The group which fermented with Gifu anaerobic medium did not show any decrease in the relative abundance of Enterobacteriaceae. Samples of the group which fermented with mare milk had the highest relative abundance of Lachnospiraceae of all the analyzed milk samples, including the T group. As anticipated, the relative abundance of Clostridiaceae_1 decreased in the groups which fermented with human milk, mare milk, infant formula USH, and Gifu anaerobic medium, and in particular, the groups which fermented with mare milk and human milk had similar decreasing trends. This was also observed in the beneficial bacterial family increase pattern between both milk types.

**FIGURE 5:**
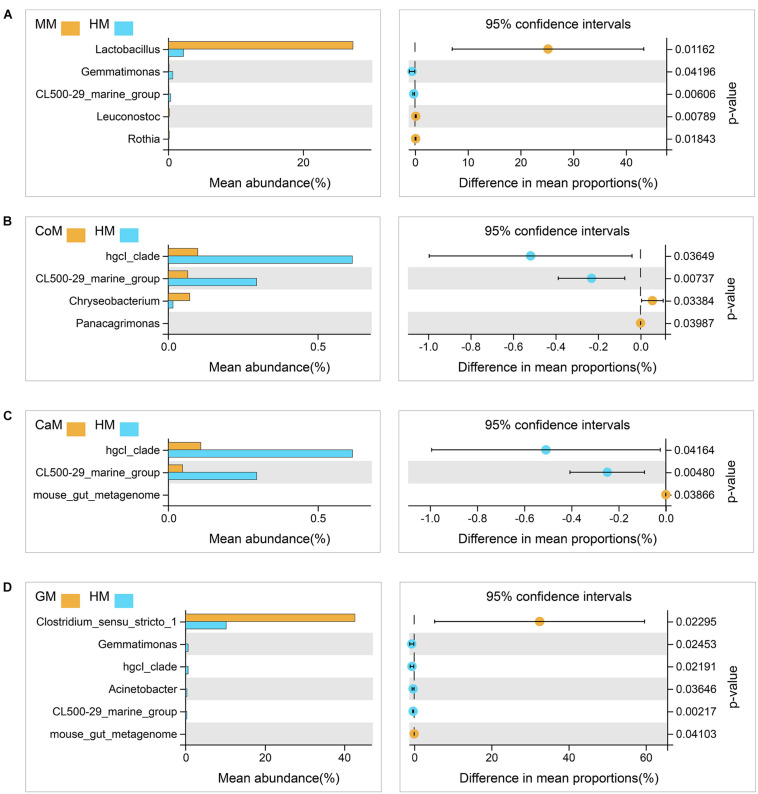
Taxa changed after fermentation. **(A)** Mare milk versus Human milk; **(B)** Cow milk versus Human milk; **(C)** Camel milk versus Human milk; **(D)** Goat milk versus Human milk. 95% confidence intervals, *p* < 0.05.

All milk fermentation groups were also compared to the T group, and the results showed that fermentation significantly altered microbiota abundance. This differentially abundant microbiota was sufficient to understand the effect of different milk on the enrichment of bacteria families. And compared with the T group, mare milk significantly increased the abundance of Lactobacillaceae and *Lactobacillus*, while the other kinds of milk significantly decreased the abundance of those (Supplementary Figure S2). Also, compared with the group which fermented with human milk, the group which fermented with mare milk increased the abundance of *Lactobacillus* significantly and had no significant difference in the abundance of *Bifidobacterium* and *Akkermansia*. The group which fermented with goat milk increased the abundance of Clostridium_sensu_stricto_1 and also had no significant difference in the abundance of *Bifidobacterium* and *Akkermansia*. Thus, mare milk was adjudged the best replacement of human milk (Figure 5).

### Correlation Analysis Between Bacteria and the Contents of the Main Components of Milk From Various Types of Animals

**FIGURE 6:**
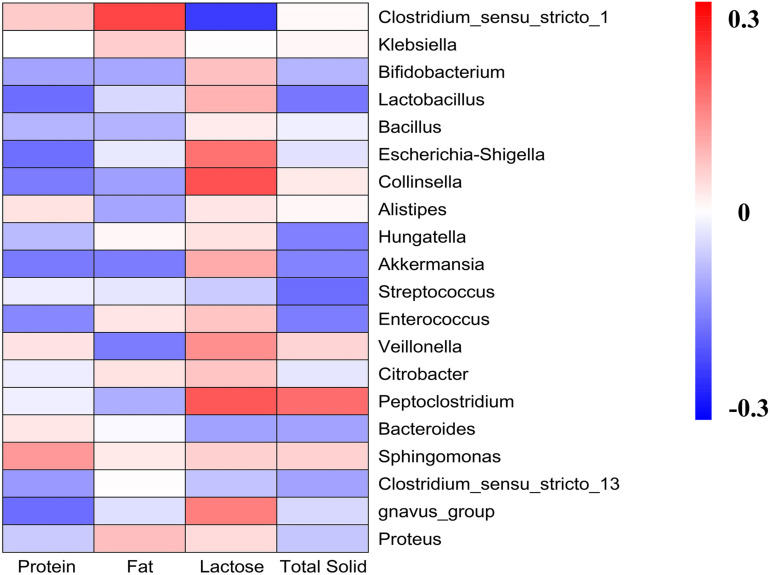
Heatmap analyses. It represented the correlation between gut microbiota and the main components of milk.

We detected the contents of the main components of different kinds of milk and correlated with gut microbiota analysis (Figure 6). Generally, results showed that the main part of milk influenced the microbiota groups known to have health-related effects, such as *Bifidobacterium*, *Lactobacillus*, and *Akkermansia*. High lactose content was related to the increasing abundance of *Bifidobacterium*, *Lactobacillus*, and *Akkermansia*, and significantly decreasing abundance of *Clostridium_sensu_stricto_1*. It was also observed that protein content was negatively correlated with *Bifidobacterium*, *Lactobacillus*, *Bacillus*, *Escherichia-Shigella*, *Akkermansia*, *Enterococcus*, and *Proteus*. While fat content was significantly positively correlated with *Clostridium_sensu_stricto_1*, and was negatively correlated with *Veillonella*, *Peptoclostridium*, and *Akkermansia*. The total solid content was negatively correlated with *Bifidobacterium*, *Lactobacillus*, *Akkermansia*, *Streptococcus*, *Enterococcus*, and *Bacteroides*, and was positively correlated with *Peptoclostridium*.

### Correlation Analysis Between Gas Pressure, pH, Bacteria and the Fermented Groups

**FIGURE 7:**
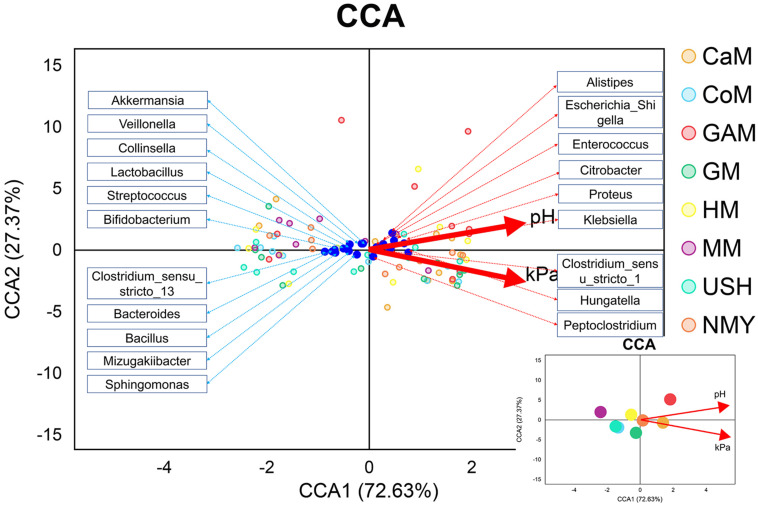
CCA analyses. The blue dots represent the gut microbiota; the red lines represent the gas pressure and pH, and the small picture represents the correlation between gas pressure and pH with groups.

We evaluated the gas pressure and pH of all milk samples and combined them with gut microbiota analysis (Figure 7). It was revealed that gas pressure and pH were positively correlated with the groups which fermented with camel milk, infant formula NMY, and Gifu anaerobic medium. These parameters were negatively correlated with the groups which fermented with human milk, mare milk, goat milk, cow milk, and infant formula USH. Gas pressure and pH also had similar effects on the distribution of gut microbial species as they were positively related to *Alistipes, Escherichia_Shigella*, *Enterococcus*, *Proteus*, *Klebsiella*, *Hungatella and Peptoclostridium*, and was negatively associated with *Akkermansia*, *Veillonella*, *Lactobacillus*, *Streptococcus*, *Bifidobacterium*, *Bacteroides*, *Bacillus*, and *Sphingomonas*.

### Potential Functional Consequences

**FIGURE 8:**
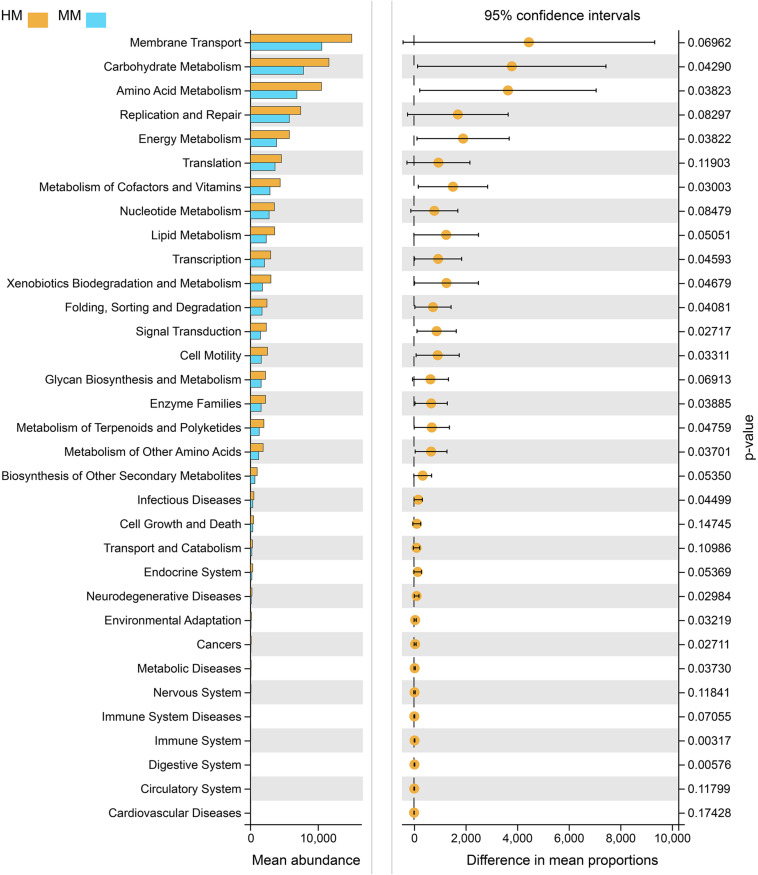
PICRUSt analyses using Welch’s *t*-test between human milk and mare milk. 95% confidence intervals, *p* < 0.05.

Functional profiling of microbial communities from the groups which fermented with human milk and mare milk was predicted using PICRUSt. Most predicted pathways were similar between the group, which fermented with mare milk and the group which fermented with human milk by the Welch’s *t*-test (Figure 8). These include metabolism (nucleotide, lipid, other secondary metabolites biosynthesis, and glycan biosynthesis), environmental information processing (membrane transport), genetic information processing (replication and repair, translation) and cellular processes (cell growth and death). These indicated that mare milk was more similar to human milk in terms of microbial communities’ functions.

## Discussion

The objective of this research was to analyze the beneficial effects of various kinds of milk on infant gut microbiota, especially the relationship between the main components of milk and the abundance of health-relevant microbiota taxa, including the abilities to improve the growth of identified microbiota, pH value, and the gas pressure due to fermentation. The main components of milk from various types of animals were determined. Although the different origin of these animal milk maybe affects their main parts, our results showed that the main components of cow milk, goat milk, camel milk, and mare milk were more consistent with those reported by Young W. Park (Park, 2017a; Park, 2017b; Park, 2017c).

The result of pH demonstrated that the effect of human milk was significantly different from other milk types, and the formula powder had the most rapid acidification ability. Total gas pressure produced by fermentation of milk depends on many factors. In terms of the composition of intestinal microbiota, considerable diversity exists both within and between individuals, and the intestinal microbiota is the source of gas pressure in the colon (Marchesi et al., 2016). Researches noted that substantial differences in gas pressure existed between individuals, which was associated with the nature of intestinal microbiota and the substrates available (Zmora et al., 2019). Gases through qualitative and quantitative analysis, provide opportunities to enhance our understanding of the intestinal function.

Moreover, while gases produced by microbiota and chemicals have been related to intestinal disorders, such as IBS and lactose intolerance (Arasaradnam et al., 2014; Berean et al., 2018; Pillai et al., 2018), there are still many uncertainties regarding the associations among intestinal gases, infant intestinal microbiota, milk, and infant health. In this study, we observed that the groups which fermented with camel milk, infant formula NMY and Gifu anaerobic medium produced the highest gas pressure after 24 h fermentation, while the groups which fermented with mare milk, human milk, cow milk and infant formula USH had the lower gas pressure. Of particular interest is that the effect of mare milk on gas pressure was similar to that of human milk. The present study demonstrated that mare milk was more beneficial to infant health, and could potentially replace human milk (at least partially).

*Bifidobacterium* is a unique bacterium in that no gas is formed as an end product of metabolism (Buchanan et al., 1975). Numerous researches assert that the increased level of the *Bifidobacterium* genus is considered beneficial because they correlate with many positive health outcomes (Hidalgocantabrana et al., 2017). *Bifidobacterium* naturally occurs in the gastrointestinal tract of healthy adults, making them a common marker for prebiotic capacity, as well as do not produce any known carcinogens *in vivo*. The concentrations of *Bifidobacterium* have a negative correlation with obesity and weight gain (Collado et al., 2008; Kalliomaki et al., 2008; Santacruz et al., 2010; Schwiertz et al., 2010). In our study, microbial diversity analyses among fecal donors seek to understand the real complexities involved in the identification of various levels among the different families. And we also observed that the level of *Bifidobacterium* was significantly increased in all groups, especially in the groups which fermented with cow milk, mare milk, and infant formula NMY, comparing the 24 h samples to the infant fecal slurry samples. J. Fotschki et al., also found that *Bifidobacterium* had a similar affinity toward mare milk (Fotschki et al., 2016). There are some researchers reported that *Bifidobacterium* and *Lactobacillus* also could improve the infant diarrheal (Saavedra et al., 1994; Guandalini et al., 2000; Chouraqui et al., 2004; Corrêa et al., 2005; Zvi et al., 2005; Roberto Berni et al., 2007). The increased level of *Lactobacillus* is judged as a beneficial effect. We demonstrated that there was also a significant increase in the genus *Lactobacillus* in all groups, especially in the group which fermented with mare milk. Results from the present study showed that mare milk enriched the abundances of *Lactobacillus*, and had no significant difference with human milk in the abundance of *Bifidobacterium*, thus the mare milk could replace human milk in feeding infants.

Recently, the possible health properties of *Akkermansia* have been gaining research attention (Ansaldo et al., 2019; Depommier et al., 2019). This bacteria was shown to correct the host metabolic disorders in obese insulin-resistant mice, is also a productive component of the intestinal microbiota in healthy humans (Amandine et al., 2013). Nowadays, interest in the role of intestinal microbiota includes various types of diseases, mental disorders (Rieder et al., 2017), neurodegenerative disorders (Westfall et al., 2017), and immune system disorders (Levy et al., 2017). In our research, the level of genus *Akkermansia* was significantly increased in the group, which fermented with mare milk, while was markedly decreased in the group, which fermented with camel milk, suggesting that mare milk may have specific beneficial effects on the overall wellbeing of infants.

Lactose is the predominant soluble digestible glycan in milk, serving primarily as a readily available energy source to newborn mammals. Findings of the present study hypothesized that the differences of *in vitro* fermentation properties of different kinds of milk on the fecal microbiota might depend on the main component of the milk, particularly the lactose content as it was positively correlated with health-related microbiota taxa. This observation aligns with a recent study investigating the influence of 2′-fucosyllactose on simulated infant intestinal microbiota (Salli et al., 2019).

Canonical correspondence analysis (CCA) model was established to analyze the correlation between gas pressure, pH, and microbiota at the genus level. Earlier, it was reported that *Klebsiella* (Costerus et al., 2012) and *Proteus* (Penner, 2015) were more likely to produce gas, while Bifidobacterium, Lactobacillus, and Akkermansia produced little or none as an end product of metabolism (Buchanan et al., 1975). It was also confirmed in our study. In the current study, mare milk fermented by infant feces had higher abundances of *Bifidobacterium*, *Lactobacillus*, and *Akkermansia* genera than those of other kinds of milk fermented by infant feces. Based on our *in vitro* study, we conclude that the mare milk is the most suitable replacement for human milk. Therefore, making infant formula based on mare milk may be more conducive to the establishment of intestinal microbiota in infants, which provides a new possibility for the study of infant formula.

Additionally, fecal samples from only ten infant donors were used in this study. Because of the individual difference in the intestinal microbiota of infant donors, a larger size of infant samples may be required to achieve a more typical view of the effects of milk from various animal types (Supplementary Figure S3).

## Conclusion

Research interests in developing a human milk substitute that would have the same effects on infant microbiota are currently on the rise. In the present study, milk from various types of animals promoted the genus *Bifidobacterium* and *Lactobacillus*, which was proportional to the lactose content of milk. Moreover, milk from mares also significantly increased the relative abundance of *Akkermansia*. Mare milk and human milk were similar, as both had low gas pressure values. Gas pressure was positively related to *Klebsiella* and *Proteus* while it was negatively associated with *Bifidobacterium*, *Lactobacillus*, and *Akkermansia*. Collectively, milk from various types of animals measured in this study display fermentability and an increase in beneficial taxa *in vitro* that have health benefits, especially mare milk, which has the potential to be a substitute for human milk at least partially.

## Data Availability Statement

The datasets presented in this study can be found in online repositories. The names of the repository/repositories and accession number(s) can be found at: https://www.ncbi.nlm.nih.gov/, PRJNA566066.

## Ethics Statement

The studies involving human participants were reviewed and approved by the Ethical Committee of Northeast Agricultural University. Written informed consent to participate in this study was provided by the participants’ legal guardian/next of kin.

## Author Contributions

NL, SW, and GH conceived the study and designed the project. NL, LZ, and GH helped to collect the fecal samples of infants and investigate infant information. NL, JS, and JG performed the experiments. NL and BL analyzed the data and drafted the manuscript. BL and SE helped to revise the manuscript. All authors contributed to the article and approved the submitted version.

## Conflict of Interest

The authors declare that the research was conducted in the absence of any commercial or financial relationships that could be construed as a potential conflict of interest.
