# Supplementary material for: Distinct Effects of Milks From Various Animal Types on Infant Fecal Microbiota Through in vitro Fermentations
Source: Front Microbiol. 2020 Sep 10;11:580931. doi: 10.3389/fmicb.2020.580931 (PMC7533598; doi:10.3389/fmicb.2020.580931)
Supplement: Supplementary file 1 [file Data_Sheet_1.pdf]

Supplementary Material

Supplementary Figures

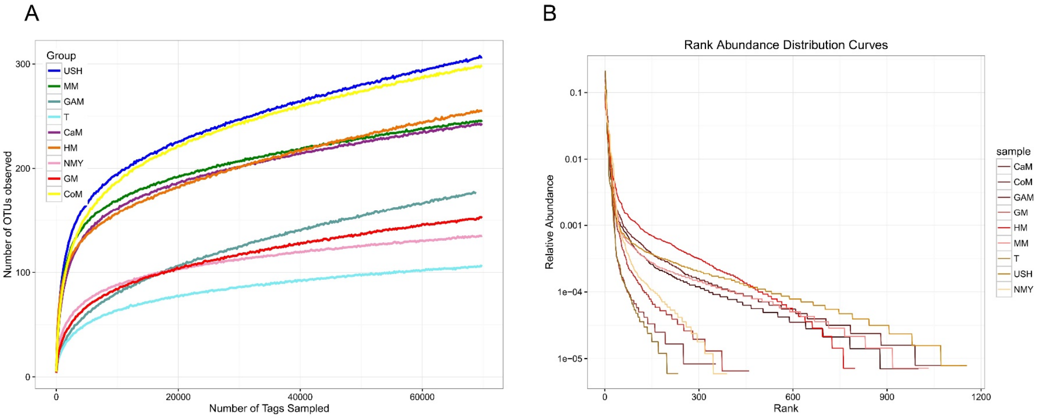

Figure S1. Species accumulation curve and the rarefaction curve.

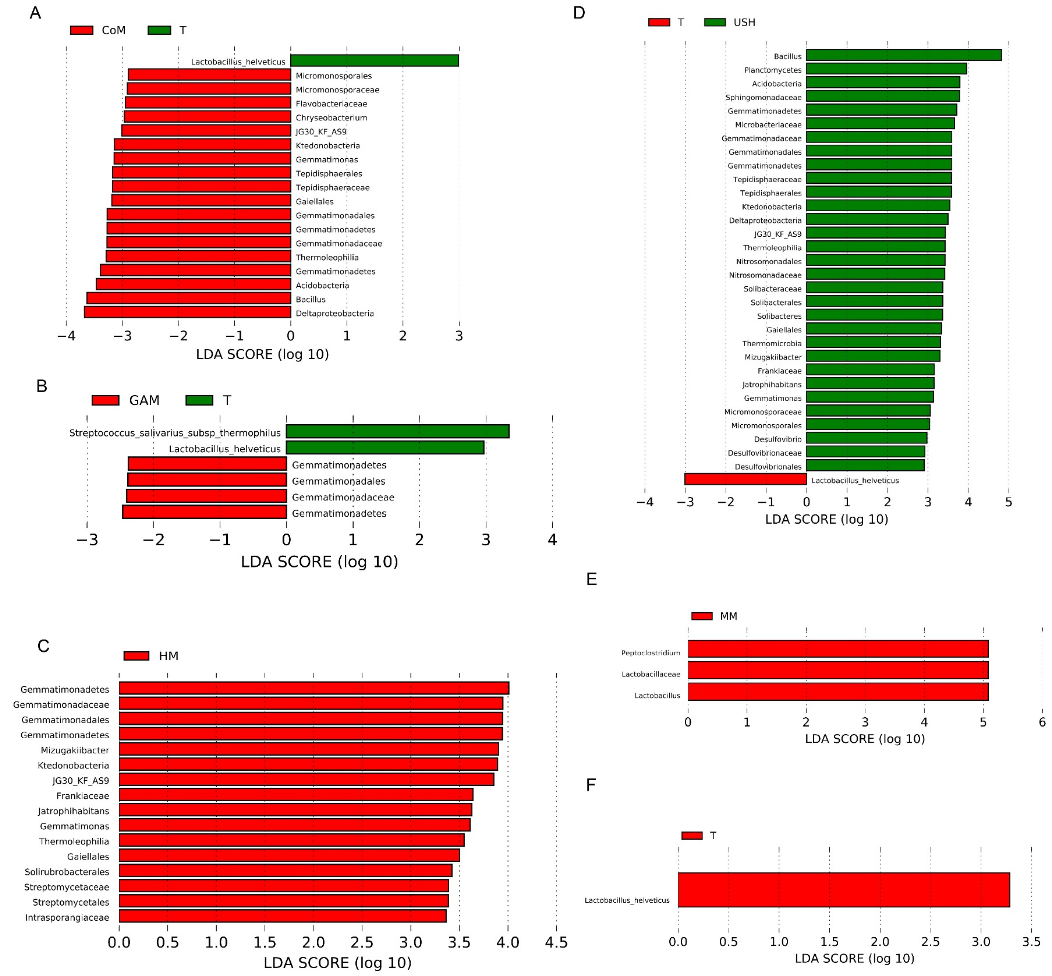

Figure S2. LefSe analysis. (A) Cow milk; (B) Gifu anaerobic medium; (C) Human milk; (D) USH infant formula powder; (E) Mare milk; (F) Goat milk. All LDA score (log 10) > 2.

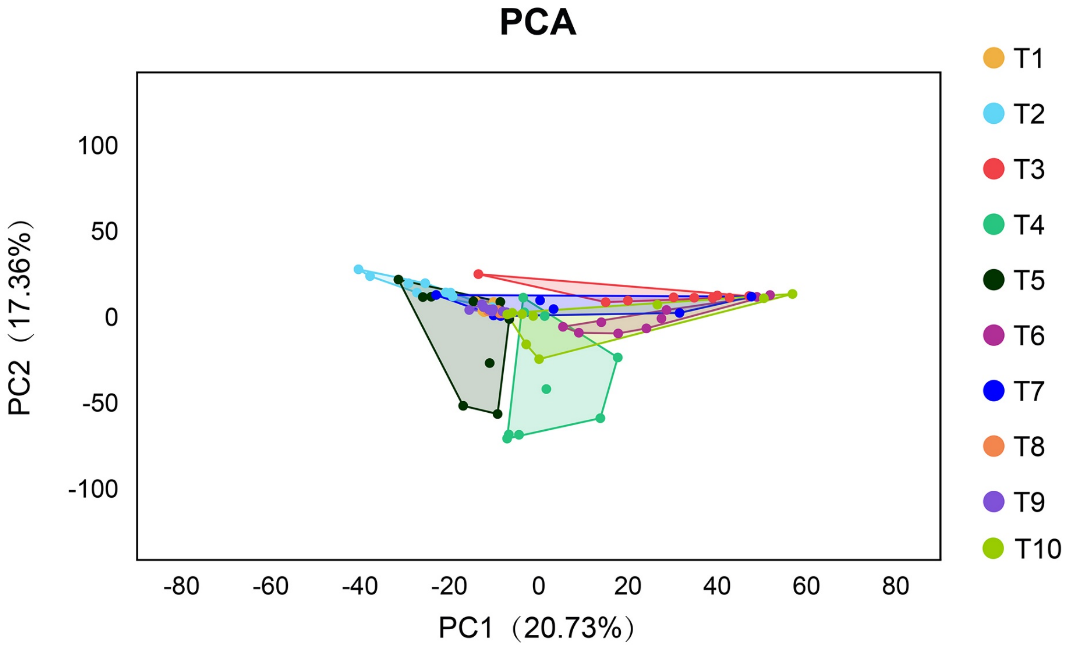

Figure S3. β-diversity principal component analysis among microbiota of ten fecal donors after 24 h fermentation.
