# Supplementary material for: Distinct Effects of Milks From Various Animal Types on Infant Fecal Microbiota Through in vitro Fermentations
Source: Front Microbiol. 2020 Sep 10;11:580931. doi: 10.3389/fmicb.2020.580931 (PMC7533598; doi:10.3389/fmicb.2020.580931)
Supplement: Supplementary file 2 [file Table_1.docx]

**Table S1.** Demographic Characteristics of Ten Fecal Donors.

|  | **Gender** | **Mode of delivery** | **Age (wks.days)**  **at time of sample collection** | **Gestational age, wks** |
| --- | --- | --- | --- | --- |
| **T1** | Male | Cesarean | 23.1 | 39.0 |
| **T2** | Male | Vaginal | 21.2 | 39.0 |
| **T3** | Female | Cesarean | 21.0 | 40.0 |
| **T4** | Male | Cesarean | 22.4 | 39.2 |
| **T5** | Female | Cesarean | 25.5 | 39.5 |
| **T6** | Female | Vaginal | 6.6 | 40.2 |
| **T7** | Male | Cesarean | 25.6 | 39.3 |
| **T8** | Female | Vaginal | 26.2 | 39.0 |
| **T9** | Male | Vaginal | 14.2 | 39.0 |
| **T10** | Female | Vaginal | 8.0 | 40.2 |
